# Operational planning for public holidays in grocery retailing - managing the grocery retail rush

**DOI:** 10.1007/s12063-022-00342-z

**Published:** 2023-01-03

**Authors:** Elisabeth Obermair, Andreas Holzapfel, Heinrich Kuhn

**Affiliations:** 1 Department of Logistics Management, Hochschule Geisenheim University, Geisenheim, Germany; 2 Chair of Supply Chain Management & Operations, Catholic University Eichstätt-Ingolstadt, Ingolstadt, Germany

**Keywords:** Grocery retail, Public holiday, Operations, Logistics planning

## Abstract

Public holiday weeks cause specific challenges in grocery retailing as sales are raising and working days for logistics processes are reduced. The paper analyzes the operational planning challenges and solutions for demand planning and disposition as well as for warehouse and transportation management of grocery retailers in public holiday seasons. A total of 22 top managers representing 20 sales lines of 17 of the top 30 grocery retailers in Germany participated in the study. Semi-structured, face-to-face interviews with logistics managers were conducted and analyzed. Uncertainties and missing resources can be identified as the two main challenges of public holiday seasons in grocery retailing. Retailers implement numerous measures that can be summarized in three categories, i.e., the adjustment of workload profiles, the adaptation of resources and modifying processes. Literature has so far considered public holidays only to a limited extent, e.g., as a parameter in forecasting models or for the application of marketing instruments. This study is the first developing a framework and providing insights into operational planning in grocery retailing.

## Introduction

**Fig. 1:**
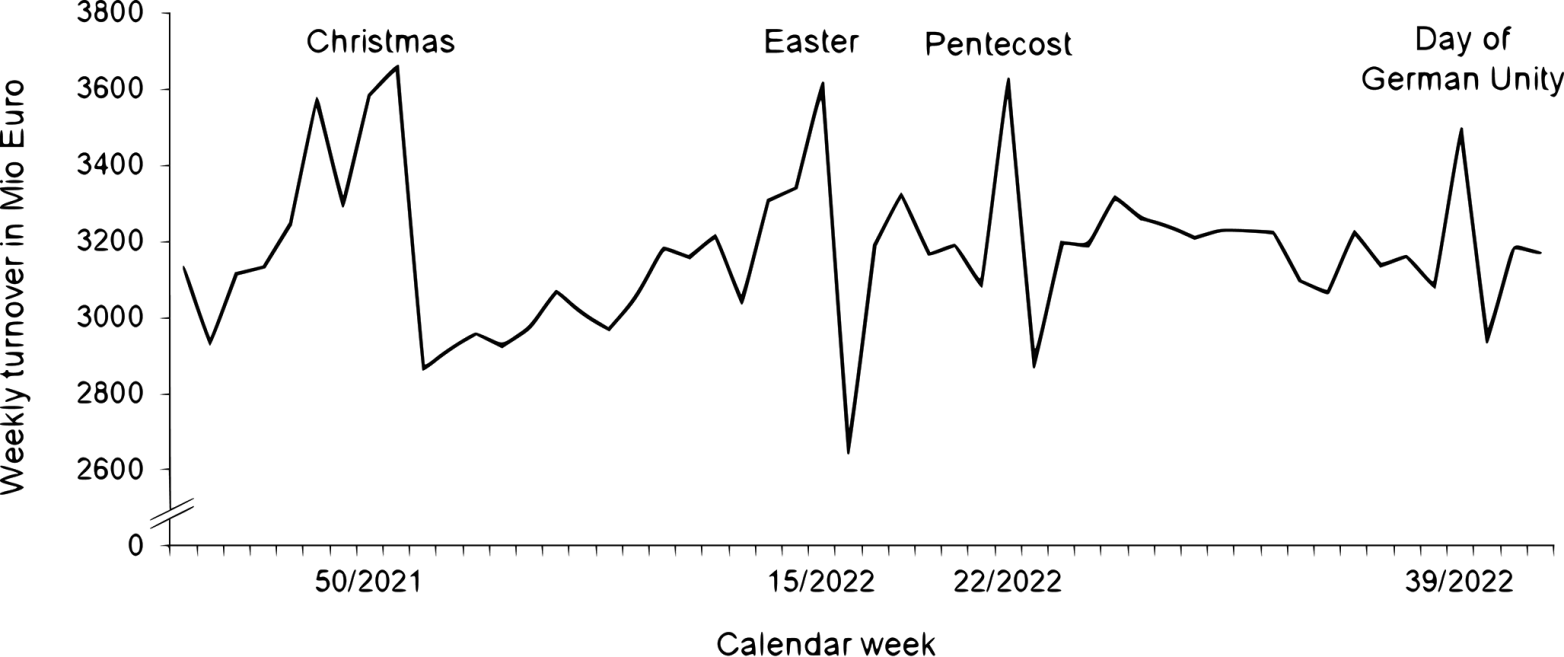
Total sales of the German grocery retail per week (Lebensmittelzeitung 2022)

In retailing, demand fluctuates during the week, the year and especially around holidays (Ehrenthal et al. 2014). Public holiday seasons are associated with an increase in revenue and play a major role for the annual performance. Therefore, retailers offer a multitude of promotional articles in holiday seasons to improve their sales (Qiu and Wenqing 2016). Moreover, instore sales per day increase during public holiday periods because customers have one or several days less for their purchases. This especially applies to grocery retailing as shown in Fig. 1 using the example of Germany in 2021 and 2022. The figure shows that sales increase noticeably at the end of the year. In calendar week 51/2021, the grocery retailers’ turnovers increased by 50% compared to an average week of the same year (Lebensmittelzeitung 2022). Consumers bought their groceries for the Christmas holidays, when the stores are closed for at least two days.

Besides sales effects, public holidays are also affecting retail operation processes. For instance in Germany, employees are not permitted to work on public holidays besides a few occupational groups such as emergency services. As a result, less time is available to perform the necessary logistical processes, but the same or even higher volumes than in average weeks have to be handled. Additionally, calendar effects change the holiday constellations every year, as several public holidays have a flexible calendar date and the ones with a fixed date are subject to weekday changes. This further challenges operational planning as historical references are often missing. Consequently, logistics plans must be updated every year resulting in a considerable additional effort. For example, during the Christmas week, several working days are omitted. This means that several delivery days are omitted and some stores can be supplied less frequently than usual due to limited transport capacity. In addition, before Christmas and New Year’s Eve a large number of additional seasonal items have to be integrated into the supply chain processes. Moreover, planning is complicated by a lack of forecast data because the available weekdays for sale vary from year to year. The changing weekday constellation of the Christmas public holidays leads to a total of seven different Christmas constellations. Sales figures from the year with the same constellation are therefore considerably out of date. The aforementioned challenges are even greater for the Easter holidays, which can be shifted by several weeks during the year. Previous year’s values cannot be carried over as the weather strongly influences the consumer behavior on a free day in spring.

In addition, some countries (e.g., Germany) have different public holiday regulations within the country, which further complicates logistics planning and operations. In the event that the distribution center is affected by a public holiday but the associated stores are (partially) not, separate delivery schedules must be created for the affected stores. In addition, these stores — if located near the state border — experience additional demand due to the holiday-shopping behavior of neighboring states celebrating a holiday. Open stores in neighboring states need sufficient stock to compensate for potential delivery shortfalls and additional demand. If replenishment authority lies with the stores, store employees must adjust their orders accordingly. These volumes accumulate in the upstream distribution centers, resulting in additional peaks.

In what follows, we refer to all the logistical and operational planning challenges in grocery retailing just mentioned as the “public holiday challenge.” Given this challenging planning conditions, a careful planning of operations for public holiday seasons is essential as retailers have to cover significant operational efforts and logistics costs (Holzapfel et al. 2016). Although there are various contributions on retail logistics and operations planning, pertinent literature so far misses a comprehensive analysis of logistics and operations challenges and suitable measures for the public holiday challenge. There are some contributions on demand planning and forecasting for high demand seasons (see, e.g., Dharmawardane et al. (2021) or Dreyer et al. (2018)) and general approaches for handling workload peaks in warehousing and transportation are presented (see, e.g., van Gils et al. (2017), Wruck et al. (2017) or Wilding and Juriado (2004)). However, the question remains open whether these general suggestions can be directly transferred given the specifics of the public holiday challenge outlined above. Additionally, retail practice urges for a systematization and structured decision support for the public holiday challenge, as we have found in pre-discussions with managers involved in logistics and operations decision making in grocery retailers. So far the various challenges are typically solved without a structured planning concept.

Thus, our aim is to identify and systematize the fields of action as well as the solution approaches of grocery retailers before and after holidays by means of an exploratory study, providing guidance on which logistics and operational measures are proven to be adequate under which conditions. We thereby contribute to literature with a first systematization and challenge-solution-framework for the public holiday challenge in grocery retailing, while retail practice can use our findings to develop their company-specific public holiday planning concept in a structured manner based on best-practices in the sector.

The remainder of the paper is organized as follows. Section 2 reviews existing contributions in the research field and formulates and motivates the research questions for our exploratory study. Section 3 then details the methodology used. Section 4 then presents the empirical findings of our study. Afterwards, Sect. 5 discusses these findings in the light of literature and demonstrates the practical implications. Finally, Sect. 6 summarizes the main results and indicates future research areas.

## Related literature

The public holiday challenges in grocery retailing influence planning and fulfillment activities at different temporal levels and in different functional areas. The aim of this section is to identify the planning areas that are particularly affected by public holiday effects. These areas form the central focus of our study. For the planning areas considered to be particularly relevant, we then examine to what extent proposals for solutions to the public holiday problem have already been developed and presented in the associated literature. Based on this we then derive our research questions.

**Fig. 2:**
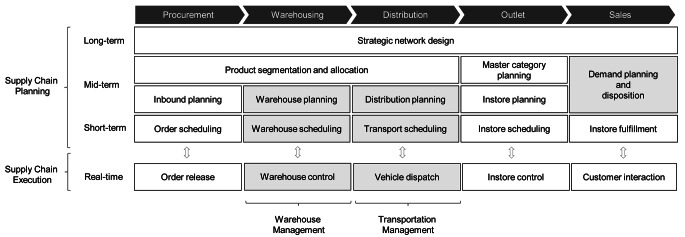
Retail supply chain planning and execution framework (adapted from Hübner et al. (2013); Meyr et al. (2015))

Supply chain planning is an intensively researched area (see, e.g., Stadler et al. (2015)). A distinction is made between three levels, depending on the temporal horizon: long-term, mid-term and short-term planning. Long-term planning makes strategic decisions that are valid for several years, such as the design of the supply chain. Mid-term planning covers the planning horizon of the next 6 to 24 months and plans the rough framework of operations in terms of volumes and resources. Seasonal fluctuations are taken into account here. Short-term planning involves decisions for a few days up to several weeks. The majority of authors describes mid- and short-term planning as operational planning area with decisions that are highly interconnected (Fleischmann et al. 2015). Given the time horizon of public holiday planning that has to be executed year by year for the different events, we focus on such operational planning tasks in the following portray of pertinent literature. Beside the temporal component, grocery retail planning can be divided into functional planning areas: procurement, warehousing, distribution, outlet, and sales (Hübner et al. 2013). While generally all of these planning areas have to be considered when arranging the retail business before public holidays, we focus in the following on mid-term sales planning (demand planning and disposition) and mid- and short-term planning problems in the areas of warehousing and distribution. We exclude procurement and outlet planning in our study because warehousing serves as a central buffer between suppliers and stores in grocery retailing and specific mid- and short-term activities resulting from the holiday effects primarily involve warehouse- and transport-related activities in addition to demand planning and disposition. This focus is also justified as these planning areas are typically seen as most critical elements of public holiday logistics and operations planning, as we have found through a preliminary study which we have conducted with experts in grocery retail supply chain management to streamline our study and to promote practical relevance (see Sect. 3.4). Figure 2 depicts the retail supply chain planning matrix according to Hübner et al. (2013) and highlights the planning areas, which we focus on.

In the domain of mid-term sales planning, Fildes et al. (2022) present an overview of the retail forecasting literature and practice including store ordering. They analyze parameters of operational forecasting which highly influence demand per stock keeping unit and store order. The demand for each item is usually calculated on a weekly or daily basis including short-term special influences. Automated store ordering systems are implemented considering individual inventory and logistics settings of each store (van Donselaar et al. 2010). Consequently, forecasting in grocery retailing requires high computing capacities and error monitoring should be focused on an operational level (Dharmawardane et al. 2021). Nowadays, choosing the right settings is ever more challenging due to shorter product life cycles and increasing assortments (Dekker et al. 2004). It is essential to understand the demand development and fluctuation of the assortment, as the calculations performed determine the order quantities and delivery times per store (Kembro et al. 2018). The exceptional demand peaks, which can be observed in public holiday seasons, are mentioned in several contributions in the literature. Dharmawardane et al. (2021) for example highlight that exceptional demand peaks are highly influenced by promotions, sales events or the weather in retailing. In this context, Alftan et al. (2015) mention that a centralization of the forecasting activities is useful for bundling information and handling the complexity of the high amount of parameters necessary. Nevertheless, Dreyer et al. (2018) pinpoint that operational forecasting can be considerably improved by analyzing promotional events, increasing the knowledge about such events. Transferred to public holiday planning, this shows the need for a deeper analysis of the challenges and circumstances of the individual holidays to be able to develop adequate solution procedures for demand planning in the respective selling seasons.

Demand planning in turn directly impacts warehouse management defining the volumes that need to be handled and thus personnel and storage capacity requirements in the distribution centers. An overview of warehouse operations problems is given by Gu et al. (2007). The focus in this domain is mainly on order picking and storage, such as the design of order picking systems (see, e.g., van Gils et al. (2018)). For picking, orders have to be sequenced and aligned to shifts enabling personnel scheduling (van Gils et al. 2018). Manual picking processes dominate in grocery retailing, because of the demand seasonality, the variability in product shape and the high investment costs of automation solutions (Petersen and Aase 2004). Storage location and assignment planning is linked to the routing of order picking, which influences picking times and costs. Operational warehouse management revises and improves the corresponding planning activities. Its effort highly depends on the complexity of the activities (Faber et al. 2013). For managing peak loads at the warehouses pertinent literature suggests balancing the volume according to the capabilities. On the one hand, the volume can be moved according to the available resources. On the other hand, the resources can be adjusted to the volume. Personnel scheduling and labor flexibility approaches are focused as main tools (Porto et al. 2019). Further on, pertinent literature frequently elaborates on the decision making process for the use of external workers when managing peak loads in warehouses (Wruck et al. 2017). However, so far there are no contributions explicitly investigating the specific conditions and corresponding solution concepts for warehouse management during public holiday seasons. As mentioned in the introduction, there are several additional factors that complicate operational planning in these seasons, besides the pure workload peaks. Thus, it is relevant to investigate the corresponding consequences for warehousing and to analyze which of the concepts that are generally suggested for handling peak loads are suitable under which conditions.

Similar conclusions can be drawn when investigating literature in the transport domain. Operational transportation planning captures the transport between distribution centers and stores and is forced to meet the high requirements of both adjacent subsystems (Hübner et al. 2013). It consists of delivery mode planning, product-warehouse allocation, transportation mode selection, and delivery pattern definition. The correlations of the aforementioned topics are discussed by Martins et al. (2018). More in-depth insights for grocery retailing are provided by Holzapfel et al. (2018) presenting a planning concept for product allocation decisions, and Holzapfel et al. (2016) as well as Frank et al. (2021) optimizing store delivery patterns. The latter affect vehicle routing striving for high vehicle utilization and minimizing driving distance and stop costs. The interface between transportation and store operation is analyzed by Kuhn and Sternbeck (2013). The packaging unit, the store delivery pattern, the lead time for store orders, delivery time windows as well as the sequence load carrier are identified as main parameters, and their mutual dependencies as well as effects on the store and transport are analyzed. However, so far it remains unclear how peak seasons like the ones before public holidays impact these planning tasks and which strategies exist to handle peak volumes in transportation, given the general transportation capacity and driver shortage. Pertinent literature notes that retailers usually purchase transport capacity from third parties to manage such peaks (Wilding and Juriado 2004), but the question remains open how this decision is integrated in the operational planning concepts and which other strategies exist.

Summarizing the findings from our literature review, there are numerous existing contributions presenting planning concepts for the standard business in retailing. There are also some contributions that provide general solution approaches for handling demand and workload peaks. However, contributions that explicitly investigate the circumstances and special characteristics of the planning situation before public holidays are missing so far. Investigating these aspects will help to provide a more concrete challenge-solution framework for the different planning tasks in operational planning for public holiday seasons. Moreover, retail practice that also misses a structured planning concept for these periods, could benefit from clear insights which solution possibilities exist for the diverse challenges and which approaches have been proven to be adequate under which conditions. In the light of this background, we can formulate the following research questions (RQ):

- RQ 1: How do grocery retailers deal with the public holiday challenge in logistics operations and why are specific strategies applied?
- RQ 2: How do context specifics in the problem settings influence their solutions?

## Methodology

For answering the research questions, we have chosen an exploratory qualitative research study. Such a study is appropriate for uncovering such unstudied research areas as data from practice enrich the theoretical findings (Stebbins 2001; Flynn et al. 1990).

### Research framework

Qualitative research is useful for studying ‘how’ and ‘why’ research questions as in our case (Pratt 2009). Using an exploratory study, we follow the grounded theory approach and develop generalizable results out of empirical data (Glaser and Strauss 1967). The methodology follows the typical structure for exploratory studies in the logistics domain using semi-structured expert interviews as portrayed in Voss et al. (2002), and has been also applied in further studies in retail logistics and operations management (see, Hübner et al. (2015), Kuhn and Sternbeck (2013)  or Upadhyay et al. (2022)).

In a preliminary study, three interviews were conducted with experts from retail logistics and relevant literature on logistics in grocery retailing was examined with regard to the public holiday challenge. The interviews lasted 60 minutes and were conducted personally with two interviewers. After these preliminary interviews the operational processes of an average week without public holidays were outlined and the terminology ‘public holiday challenge’ was clarified. All planning areas in retail logistics (see Fig. 2) were systematically queried and the influence of holidays as well as the challenges were asked. The planning areas were thereby structured in procurement, warehousing, distribution, outlet and sales, as commonly applied in retail logistics research (see, e.g., Kuhn and Sternbeck (2013), Hübner et al. (2013) or Dreyer et al. (2018)). With the help of the pre-study and our literature review, we identified the mid- and short-term planning levels as well as the retail business functions demand planning, warehousing and distribution as most affected areas. We decided to focus our study on these issues to limit the scope and to capture the respective problems in detail. In-store logistics was considered, in case being directly connected with another issue of the focus areas, as most retailers organize instore logistics in separate divisions and thus additional interview partners would have been necessary. The insights gathered from the preliminary interviews and literature research were used as a structural basis for developing an interview guideline.

### Target group

Grocery retailing is characterized by country-specific patterns and shopping behaviors (Hübner et al. 2016). Creating a unified context and achieving in-depth insights, the focus of the study is on the German grocery market (Voss et al. 2002). The German market shows specific characteristics compared to other European countries or, for example, the USA. Germany has a large number of public holidays on which work is generally prohibited. This also applies on Sundays, when the stores are closed and no logistical processes can take place. In addition, the constellation of public holidays changes in terms of calendar and/or working day and may differ in the federal states within Germany. These parameters limit retailers and face them with a particular challenge. The unified context, i.e., the German grocery market, enabled us to compare the participating companies and to evaluate the representativeness of the recorded data. The 30 top-selling grocery retailers in Germany were the target group of our study (see Retailytics (2018)).

In total 20 sales lines of 17 of the top 30 German grocery retailers took part in the study, resulting in a participation rate of 57%. This high response rate demonstrates the importance of the topic. The participating sales lines cover around 70% of the market volume, which supports the external validity and strengthens the representativity of the study (Bryman and Bell 2015). Table 1 shows that our study involves all sales formats and a large variety of company sizes.

Within the companies, we selected the interviewees according to the criteria, that they have a holistic view of the logistics structures and processes of their company (Manuj and Pohlen 2012). This enables them to assess the public holiday challenge across all areas considered. Therefore, all interviewees have an overall responsibility for logistics and supply chain management within their company. In total 22 top managers were interviewed. Table 1 summarizes their positions.

**Table 1:** Overview of participating companies and interviewees

**Sales format**	Full-range provider	Discounter	Wholesaler	Specialized retailer
Number of participants	10	5	3	2
**Number of stores**	<=500	]500; 1,000]	> 1,500	
Number of participants	10	4	6	
**Position of interviewee**	Board member	Division manager	Department manager	
Number of participants	2	9	11	

### Protocols

For our exploratory study, we designed an interview guideline with a flexible order of questions as suggested by Manuj and Pohlen (2012). The objective was to create an open conversation with free answers. This should establish the necessary level of trust. Our interview guideline was tested in a pre-test interview to check how the questions are interpreted and to strengthen the validity and reliability (Flynn et al. 1990). Moreover, the interview guideline was continuously modified during the data collection to improve our growing insights of the challenges studied (Manuj and Pohlen 2012). Prior to interviewing, each interviewee received an overview of the questions to enable preparation. This overview can be found in the appendix.

### Data collection

Data of the study were collected by conducting semi-structured, face-to-face interviews with logistics experts following a multi-step research design (Manuj and Pohlen 2012). It is an effective way of obtaining in-depth information. The same two interviewers conducted all interviews at the retailers’ offices, except two interviews, which were conducted by telephone because of time reasons. The average interview duration was 72 min. We wrote field notes during the interviews and compared and discussed them directly afterwards to check the right understanding and to achieve internal reliability (Bryman and Bell 2015).

After a first round of invitations, nine of the top 30 grocery retailers in Germany participated in the study. As we gained more insights after the analysis of each interview and noticed that there are substantial differences in processes, structures and solutions between sales formats, we decided to proceed and to investigate each sales line of the retailers separately. Additionally, we sent a reminder to the retailers already invited. Another six participants were gained. The analysis and coding of the last interviews of this group further enriched our findings only to a limited extend. However, to insure reaching the saturation level (Glaser and Strauss 1967), we sent out a third round of invitations to those retailers that had not answered so far. Two additional retailers participated, but we could not add any new code or category to our analysis. Thus, we reached the theoretical saturation level after a period of six months, and stopped the data collection.

### Data analysis

The data analysis follows grounded theory, in which theory should be built by developing categories and using a constant comparative approach (Glaser and Strauss 1967; Manuj and Pohlen 2012). For this aim, an inductive analysis was applied by formulating abstract groups and assigning the data to them (Gephart 2004). By coding the data, we built substantive categories as the categories are ‘descriptive’ (Maxwell 2008). The coding was done by two researchers using the qualitative computer analysis program MAXQDA and working independently to reach internal reliability (Bryman and Bell 2015). Thereby, each code was assigned to a phrase of the interview notes such that the codes could be compared and discussed in the research team. This was done in regular meetings. There we also analyzed, whether and which new insights were gained from the respective interviews. Data were conducted and coded until the theoretical saturation was reached as described above (Glaser and Strauss 1967).

For structuring the data, we used the Gioia method (Gioia et al. 2012). Thereof, 944 relevant phrases resulted, which we assigned to codes. Finding similarities between the codes, we built 332 first order categories. In the next step, the first order categories were classified in 97 second order categories to get the more generic dimensions. They depict the single sub-problems and the solution activities of the retailers facing the public holiday challenge.

## Empirical findings

This section presents the main findings of our study. It focuses on demand planning and disposition (Sect. 4.1), warehouse management (Sect. 4.2) and transportation management (Sect. 4.3).

### Demand planning and disposition

Demand forecasting plays a major role as it affects all further planning areas. It is essential for the availability of goods in stores and thus for the sales optimization before public holidays.

Transparency about forecasting accuracy in public holiday seasons is not always available and varies greatly between the retailers interviewed. While some retailers report forecast accuracies of only 35% in public holiday seasons, others manage to produce quite accurate forecasts with only 5% average deviation.

#### Holiday logistics challenges

Three main challenges emerge from our analysis. There is a high additional effort for manually adapting forecasts. This effort is further increased by the weather influencing the shopping behavior. Finally, short-term ordering behavior by the stores disturbes smooth disposition planning.

*Manual forecasting effort.* Public holidays require a separate forecasting process which comes along with additional manual calculation and adaptation effort. This is reported as a special challenge by 25% of the participating retailers. Many retailers have problems to implement seasonal effects in their IT systems or the complexity of the forecasting systems would not stay manageable. Consequently, manual planning of public holiday weeks is necessary, which is time-consuming. Previous or comparison years’ values often can only serve as a starting point for public holiday forecasts due to a large number of changing or unknown parameters, e.g., the increasing number of stores, the overlap with school holidays that changes year by year, or the offers of competitors which are only limited predictable. Additionally, more promotional articles than usual are offered in public holiday seasons and there is a considerable number of product innovations every year. Forecasting for these new products is difficult since historical data are missing.

*Forecasting demand for public holidays is very time consuming. We work availability-oriented, what means that the availability of products in the stores is more important than a possible loss, if we have to throw away surplus goods.* (Discounter 2, Department manager).

*Weather influences on shopping behavior.* The weather forecast is crucial for how customers plan their free days and thus their holiday purchases. Previous years’ sales therefore cannot generally serve as a meaningful basis. In addition, weather forecasts are not included in automated demand planning processes at most retailers. As a consequence, the weather forecast has to be considered by short-term manual adjustments. This explains why approximately 35% of the grocery retailers indicate that the weather volatility noticeably complicates demand planning in holiday seasons.

*Forecasting demand for public holiday seasons is very complex. Precise planning is often only possible at short-notice because of the weather.* (Wholesaler 1, Division manager).

*Short-term ordering behavior.* Weather volatility and little long-term planning effort lead to shortterm ordering by store managers. This ordering practice is known to be a major factor causing out-of-stock situations (Moussaoui et al. 2016). 25% of the retailers indicate short-term ordering behavior of their store managers as a special challenge for the holiday season disposition.

However, several retailers allow their store managers very short ordering times on a regular basis, even before public holidays, to achieve high customer service levels. This requires high stock levels and flexible personnel resources in the distribution center.

*80% of our stores reorder before public holidays, what is possible at short notice for the next day.* (Full-range provider 2, Division manager).

#### Solution concepts

Grocery retailers differentiate between the standard assortment and promotional products when developing forecasting solutions for public holiday seasons. The standard assortment includes all regular and seasonal articles, such as baking ingredients during the Christmas season. Promotional items are listed for a certain period of time such as chocolate eggs before the Easter holidays. Moreover, retailers declare regular products that are included in advertising campaigns during the holiday season as promotional items. For both the standard assortment and promotional articles dedicated solution concepts exist.

*Standard assortment.* Basic store order proposals for the standard assortment for public holiday weeks are generated by IT systems at all interviewed retailers as rich data sets from standard weeks are available. For two thirds of the participants, the proposals are based on the previous year’s figures as these are assessed as most meaningful. The remaining retailers include further past values or use data from the year with the same weekday constellation of the public holidays.

*The IT system considers the calendar constellation of public holidays. For example, the sales before a public holiday at the beginning of a month are higher as the customers have more money available.* (Discounter 1, Department manager).

Half of the retailers mostly rely on these automatically generated order proposals, thus stores should maintain their stocks carefully. This automated procedure is, however, not applicable for the complete standard assortment. It is particularly difficult for goods sold by weight, such as fruits.

The other half of the retailers apply manual approaches to forecast their standard assortment in public holiday seasons. This means, order proposals by the IT system are fundamentally revised manually as an accurate parameterization of the system gets too complicated and the results are too imprecise. 50% of this group of retailers centrally quantifies these figures and pushes the associated product volumes into the stores without consulting store managers. Working at capacity limits and the high complexity of logistics processes during the holiday business are reasons why short-time orders of store managers are banned. In addition high coordination effort results. Nevertheless, the other half of retailers explicitly bases their predictions on the experience of store managers because of their knowledge of local customer behavior.

*Store employees use an action planner to plan the seasonal articles, especially for the dry assortment. They decide when they want to reach the maximum storage and how they can avoid large order quantities.* (Discounter 2, Department manager).

To limit the forecasting effort, some retailers define a limited number of critical articles that have a major impact on sales or that have caused problems in previous year. Only for these products, they adjust the system proposals manually.

*We define corner articles. That are around 300–500 products out of 100,000. The automated forecasts for these corner articles need to be modified manually. We are therefore contacting the stores and plan the stockpiling.* (Full-range provider 10, Division manager).

*Promotional articles.* Retailers organize demand planning and allocation of promotional items quite differently, i.e., centrally or decentrally.

40% of the interviewed retailers, among them 80% discounters, have centralized the forecasting of promotional articles. Centralization leads to a more efficient and precise forecasting process as central marketing initiatives and long supply times could be better incorporated. The latter one is especially relevant because non-food import products are mostly ordered up to one year in advance. In addition several manufacturers only produce on demand. The responsibility for these tasks varies between the retailers interviewed.

*Promotional articles are allocated by the logistics department as centralization is easier and enables a better disposition for the sales department.* (Discounter 1, Division manager).

60% of the retailers rely on a decentralized demand planning and allocation of promotional articles. The sales department makes a pre-selection and the store managers order their desired quantities. They use advance inquiries to avoid short-term orders and to be able to place the orders early at the manufacturers, thus increasing the chance of receiving the delivery in time. Additionally, the order lead times for the stores for promotional articles are extended depending on the merchandise category to be able to handle the order volume fluctuations due to the decentralized disposition.

*Summary.* In holiday seasons retailers predict and dispose regular and promotional products quite differently. Regular products are forecasted and replenished by data-driven approaches if appropriate past-data are available. Alternatively, critical products of the standard assortment that feature high demand volatility are manually disposed. Promotional non-food articles are mostly forecasted and disposed on a centralized level in particular if these articles are sourced from afar. Retailers apply decentralized forecasting and disposition systems if local consumer behavior gains significance.

### Warehouse management

The grocery retailers interviewed distribute on average 81% of the entire product volume sold via their own distribution centers. 50% of the retailers handle at least 95% of their product volume via DCs. This gives warehouse management a special significance.

#### Holiday logistics challenges

Three main issues challenge warehouse management in public holiday seasons: over utilization, delayed incoming goods, and shortage of appropriately skilled employees.

*Over utilization.* In public holiday seasons the warehouses are running at their capacity limits. 65% of retailers report that warehouses actually are over capacity. Goods, for example, are already stored in the corridors. Especially for full-range providers this problem is prevalent due to their broad assortment. Running over capacity causes several safety and performance issues in distribution centers. Pallets stored in the corridors increase the risk of accidents and blocking situations as pickers and forklift drivers move in the same corridors. The high amount of employees working simultaneously in addition causes a shortage of warehouse utilities. Moreover, picking ways increase as pallets with the same goods are distributed over several corridors due to missing storage spaces. Furthermore, the high amount of promotional items in public holiday seasons causes additional performance issues as their handling is not as trained and efficient as for standard items. Thus, a considerable amount of extra hours has to be planned, which further increases capacity utilization.

*Forklift drivers and order pickers are in each other’s way. […] A doubled number of employees in the warehouse does therefore not mean a doubled output, but rather a loss of performance per employee and an increase of the stress level for everybody.* (Full-range provider 5, Division manager).

*Delayed incoming goods.* Delivery delays of incoming goods are common before public holidays for several reasons. The high order volumes are challenging for the suppliers and they therefore often struggle to manage the respective production volume in time. Delays are further caused by scarce transportation capacities. In addition, traffic jams around holidays postpone deliveries. Delayed deliveries are especially critical if fresh products or items with large order volumes are affected.

*Shortage of skilled employees.* A significant amount of retailers interviewed (35%) emphasizes a shortage of skilled employees in public holiday seasons. However, a sufficiently large employee base is necessary to handle the increased volumes and the high number of additional promotional articles. Furthermore, the pressure in distribution centers before public holidays is high and error-free work is particularly important. Thus, skilled and experienced employees are required. Permanent employees alone are, however, typically not sufficient to handle the peaks. Thus, additional temporary workers are necessary. Their availability is, however, limited as temporary employment agencies are just as affected by skill shortage as the retailers.

#### Solution concepts

Grocery retailers apply spatial, organizational and personnel measures, and they adjust workload profiles to handle the warehouse challenges occurring in public holiday seasons.

*Spatial and organizational measures.* Seasonal and promotional items require considerably additional effort in warehouses. The majority of retailers interviewed (80%) separates promotional articles spatially from the regular assortment. They place these items in separate locations within the warehouse or even use additional warehouses for distributing. Experienced workers are responsible for picking and packing as often special handling is required and promotional displays have to be built.

*We do not try to pick the promotional items for the holiday business together with the everyday business. They are separated in time and space. For example last year, a single, very experienced employee has exclusively picked and packed the displays for Christmas on our warehouse gallery from October to December.* (Wholesaler 1, Division manager).

To handle the additional volumes of regular items in holiday seasons, retailers create buffer locations or separated areas at the warehouses as far as possible. From an organizational point of view, warehouse planning prioritizes outgoing product flows as a continuous store delivery is necessary to ensure the availability of goods. On the inbound side, a close cooperation with suppliers enables just-in-time deliveries, which in turn saves storage room in the distribution center. A good supplier relationship further helps to reduce the risk of delivery failures. Several retailers foster better supplier relations by paying a higher purchasing price to suppliers in public holiday seasons. Additionally, small suppliers with little market power struggling with missing transport capacities, are supported by the retailers organizing the respective deliveries.

*Personnel measures.* Retailers try to match personnel capacity to demand as close as possible. More than half of the retailers interviewed plan by default a build up of overtime before public holidays. This is enabled by flexible working time models. Shifts are extended up to ten hours and additional shifts are added, for example in the night or on Saturday. Weekend work is generally organized on a voluntary basis at most retailers and mainly the best-before-date-critical product groups are picked.

*Employees have ten hours days. This is agreed with the workers’ council already in August for Christmas, for example.* (Full-range provider 5, Division manager).

Retailers announce a ban on taking leave in peak seasons to guarantee that the maximum number of permanent employees is available at these times. They only approve vacation inquiries in exceptional cases. Nevertheless, retailers prioritize employee friendly solutions and offer further benefits to avoid employee fluctuations in times of skill shortage and enhance their attractiveness for new potential employees. For example, retailers announce staff schedules early and establish company kindergartens.

*Especially before Christmas, there are holiday quotas. However, we try to release employees with children, who rely on the vacation times, over the busy days, as well.* (Discounter 3, Department manager).

Especially, before holidays with more than one day free, e.g., Christmas or Easter, the own personnel resources, however, might be insufficient to handle the additional volumes. In these cases retailers contract temporal workers.

*At Christmas and Easter, we have ban of leaves, recruit high school college students and the shifts start at 5 am instead of 6 am.* (Discounter 2, Department manager).

*Adjustment of load profiles.* Shifting the workload to less busy periods is in addition to the measures named before another key concept to respond to the peak load in the holiday season. On a mid-term planning level retailers use pre-picking and stockpiling at the stores to balance distribution center workloads. For example, retailers begin to build up the holiday ambient assortment for Christmas already in September/October. Before single holidays, picking and deliveries of the ambient assortment are only adjusted up to four weeks in advance. Within the ambient assortment, slow-moving items with a low value density are considered first, followed by fast-moving items and items with a high value density. The delivery volumes of perishables, however, are increased just one or two days before public holidays. Moreover, 40% of the retailers interviewed move promotion campaigns into less busy times.

*The purchasing department determines for which key articles the stock ranges are adjusted because of the financial commitment. 15,000 pallets could be handled four weeks earlier at Christmas.* (Full-range provider 9, Board manager).

Short-term, 85% of the retailers switch orders in shifts with free capacities. More than half of the retailers pick orders in an earlier shift to balance the capacity load, especially for the ambient assortment. The pre-picked orders are stored in the distribution center or in trailers until delivery.

*Trailers are pre-loaded although it is not yet clear which service provider will actually drive the tour. The trailer is then parked in the yard.* (Specialized retailer 2, Department manager).

Additionally, 10% of the retailers pick fresh products, fruits and vegetables on Sunday, in the night or on the public holiday itself. This facilitates goods processing, but results in significantly higher personnel costs and is regulated strictly by law.

*Summary.* Most solution concepts presented especially target the main challenge of warehouse management which is the capacity shortage. Delivery delays are faced by stockpiling and cooperations with suppliers, while the skill shortage is tackled by providing attractive staff benefits. Generally, a close cooperation with sales and purchasing departments as well as stores and suppliers facilitates adequate warehouse operations in public holiday seasons and offers opportunities to balance capacities.

### Transportation management

Transportation between distribution centers and stores significantly contributes to total logistics costs in grocery retailing (Holzapfel et al. 2016). Retailers, however, organize their transportation processes quite differently. 40% of the interviewed retailers outsource the transport to one or more long-term contracted third parties. 20% of the retailers operate an own fleet to reduce the risk of delivery failures. The remaining retailers (40%) manage on average half of their transport volumes themselves and subcontract the remaining share.

#### Holiday logistics challenges

The general lack of drivers constitutes a big challenge for transportation management already in standard weeks. The high additional volumes in public holiday seasons and the omission of delivery days due to the holidays as well as frequent traffic jams challenge transportation management further. Therefore, it is not surprising that the lack of transportation capacity, driver shortage and the complexity of transportation tour planning are the top challenges named by the participating retailers.

*Lack of transportation capacity.* Free transport capacity is rather limited in Europe and especially in Germany. In public holiday seasons this limitation gets particularly relevant. The demand increases and fewer delivery days are available. Consequently, the daily transport volumes substantially increase. Half of the retailers interviewed name the lack of transportation capacity as the greatest challenge in public holiday seasons.

*The greatest challenge are the missing transport capacities in public holiday weeks. This problem […] affects the entire supply chain. It cannot be solved ad-hoc as we have of course optimized our plans in the past, too.* (Full-range provider 8, Division manager).

Frequent traffic jams additionally restrict the usage of transportation capacities. Delivery tours last longer and fluctuate more in those times.

*Truck drivers for the afternoon/night tour have to wait in the distribution centers at the beginning of their shift if the trucks from the morning tour are stuck in traffic jams.* (Discounter 2, Department manager).

*Shortage of drivers.* Many German retailers work with employees from Eastern Europe to face the general shortage of drivers. These employees, however, have general agreements to visit their home countries on the bridging days around public holidays. Retailers provide these benefits to gain drivers’ company loyalty. This commitment, however, increases the shortage of drivers especially around public holidays. In addition, drivers’ salaries increase along with driver shortage, which is a great challenge in grocery retailing due to the comparatively low profit margins.

*We need a solution for the transport. We cannot continue like this as there is hardly any capacity left. Truck drivers are already attracted with a premium of three to five thousand euros.* (Full-range provider 8, Division manager).

*Complexity of holiday tour planning.* One quarter of the retailers emphasize the effort for designing special delivery tour plans around public holidays. Stores are often unable to process the increasing volumes at the delivery days as they do not have enough storage space and/or employees to receive the goods. Delivery quantities and frequencies therefore have to be aligned on each other.

*Tour adjustments are a big challenge. In total, no delivery day should be canceled; only those deliveries that lie on the holiday itself should be postponed. This is difficult and must be planned in the long-term.* (Full-range provider 7, Department manager).

Moreover, tour time fluctuations are more likely around public holidays due to the intense traffic volume and thus, tour planning gets further complex. Various retailers also note in this context that the compliance to the working time act is challenging in those situations. Driving times and rest periods for drivers are defined by law. Thus, on the one hand, tour planning has to include time buffers for traffic jams. On the other side, the grocery retailers have to ensure a high utilization of their resources due to the lack of transportation capacity and drivers. Driving bans on Sundays and public holidays as well as local restrictions on night deliveries, vehicle weights, and store access times additionally complicate the building of feasible delivery tours.

*One of the biggest challenges is the high complexity of the creation of delivery plans. Under consideration of the public holidays and all the other limiting factors, we try to balance the peaks and to work within capacities.* (Specialized retailer 1, Division manager).

The federal structure of Germany further complicates delivery tour planning. It may happen that a distribution center is closed and some of its associated stores are open because they are located in a state not having a public holiday, and vice versa.

#### Solution concepts

The retailers interviewed adapt transportation capacities and adjust delivery schedules to respond to the challenges in public holiday seasons.

*Adapting capacities.* 45% of the retailers interviewed plan more trucks and tours for the public holiday business. Regular tours are separated to more vehicles or the same tours are run multiple times. Thus, retailers plan for an increased capacity usage but assume a higher volatility of its usage.

75% of the retailers without an own fleet reserve additional transportation capacities early in advance at their third party providers, or they mention this additional option in the annual contracts. This ensures an equivalent price for the potentially additional capacity required. Half of these retailers make a tight calculation to limit the risk of overbooking and buy additional capacities on the spot market. However, before public holidays prices can even increase up to 50 to 60% compared to standard weeks.

*We reserve trucks before public holidays as it is hard to get anything on the daily market. But if we have booked too much, then high costs arise. For example Easter 2018, we had ten trucks for three weeks too much due to an incorrect forecast. *(Full-range provider 9, Board member).

Retailers operating their own fleet typically avoid to make recourse to spot markets. They apply different measures to ensure sufficient capacity in public holiday seasons. They, for example, switch capacities between delivery regions, or they offer their drivers special benefits, like luxuriously equipped trucks or company kindergartens to foster drivers’ loyalty.

*Adjustment of delivery schedules.* Besides capacity measures, retailers adapt their delivery schedules in various ways to manage increasing transportation workload during public holiday seasons. In most cases they increase the tour density compared to standard weeks to save capacity. Thereby, 35% of the participating retailers adapt the designed tours of their systems, while nearly half of the retailers (45%) manually elaborate their special tour plans on the basis of predefined standard tours. The remaining 20% of the participants outsource the tour planning to third parties.

Retailers operating with a heterogeneous store structure classify their stores according to size and number of delivery days. They then apply homogeneous adaption strategies to all stores of one category.

*Store groups are created that have the same delivery days, such as Monday, Wednesday and Friday. We decide for the whole group, whether a delivery day should be canceled or not.* (Full-range provider 4, Department manager).

Retailers increase night deliveries to those stores, which are equipped with non-personnel storage rooms. This avoids traffic jams and saves capacity during the day shift. Furthermore, 25% of the retailers interviewed extend delivery time windows to increase the flexibility for tour planning.

Nevertheless, most retailers have to increase the number of delivery days to handle the additional volume, or they have to switch the regular delivery day because this just constitutes the public holiday. In the latter case, retailers move all delivery days in the week before a holiday one day on-wards (pre-delivery) or all deliveries after the public holiday one day backwards (subsequent delivery).

Furthermore, 35% of the participants emphasize that they adjust tour plans already many weeks before a public holiday. This enables pre-deliveries ensuring the product availability in the stores while balancing capacity peaks. The increased delivery volumes can therefore be spread over several deliveries. Moreover, transport capacity in the public holiday week is then available for prioritized product groups like fruits and vegetables. As these adjustments directly affect store management, stores have to be involved or at least informed timely. However, 20% of the retailers (full-range providers and discounters) emphasize that stores are not involved in pre-delivery decisions because of complexity reasons.

Finally, there are several full-range providers that increase direct deliveries from the suppliers to the stores at Christmas or Easter seasons. This is done particularly for promotional items and displays that would lead to high extra capacity requirements at the retailer. Thus, transportation is outsourced to the suppliers and the retailers save scarce capacity.

*Summary.* Retailers apply several measures to manage the additional transportation volume and the missing delivery days in public holiday seasons. They increase transportation capacities and adapt their tour plans in many ways. In addition, they pre-deliver stores, apply night deliveries and extend delivery time windows to be able to use the standard tours as far as possible.

## Discussion

**Fig. 3:**
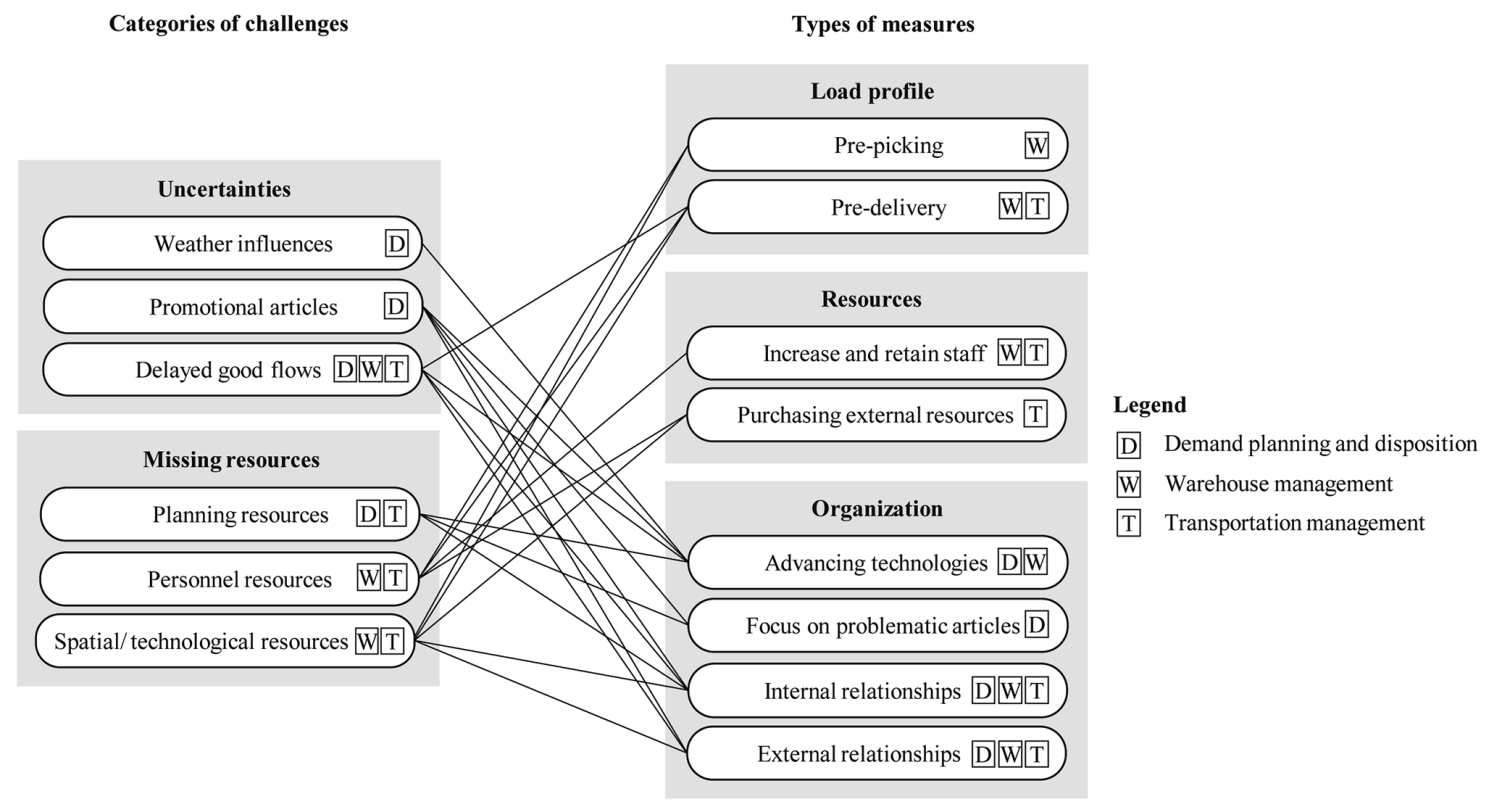
Categories of challenges and types of measures identified

Our study discloses two main reasons that cause particular challenges in grocery retailing within public holiday seasons, i.e., uncertainties and missing resources. In addition we find that retailers take up these challenges by a divers set of measures, which can be categorized into three main approaches, i.e., adjustment of workload profiles, adaptation of resources, and modifying processes and organization. Figure 3 lists the main challenges and measures, and it additionally shows which measures are used by the retailers to face one or more of the individual challenges.

In the further course of this section we compare the main challenges and solution approaches detected with the results already available in literature. Additionally, we portray the links between the planning areas and derive the managerial implications of our findings. In doing so, we highlight the theoretical and practical contribution of our study.

### Holiday logistics challenges in the light of literature

*Uncertainties.* As known from previous contributions in literature demand generally fluctuates due to uncontrollable uncertainties such as the weather, short-term orders or promotional articles (Taylor and Fearne 2009). System and physical inventory figures vary on average by 35% (Moussaoui et al. 2016). Several retailers report for example sales peaks of additional 50% per day.

The literature develops demand forecasting models considering public holidays or the weather as external parameters (see, e.g., Gur Ali and Pinar (2016)). However, the influence of the weather on the demand before public holidays in grocery retailing has not yet been analyzed. Furthermore, scientific papers mostly classify public holidays and shopping events, e.g. ‘back to school’, as one group. Thus, public holidays are studied with regard to promotional items and their pricing from a marketing perspective (see, e.g., Qiu and Wenqing (2016)). According to Taylor and Fearne (2009), fluctuation of food demand is more often caused by advertising activity than by seasonality or weather conditions. Nevertheless, we show that the seasonality and the current weather situation leads to less accurate forecasts around public holidays – especially for promotional articles. Our study also shows that the respective weekday constellation highly influences customer shopping behavior and has to be considered when forecasting public holiday demand.

Uncertainties in public holiday seasons also effect logistics planning. Henao et al. (2015) already note that employing the actual number of employees required is difficult when volumes fluctuates. Our study confirms this finding and adds that this also is valid when reserving transportation capacities. Transportation capacities, however, have to be contracted long-term or otherwise have to be bought at considerable higher prices on the spot market.

The transport capacities required are not only influenced by an increased holiday demand but also by intense traffic in public holiday seasons. During these periods, delivery times fluctuate and deliveries from suppliers are delayed. In grocery retailing these delays are generally frequent and considerably impact the operating profit (Sanchez-Rodrigues et al. 2010).

*Missing resources.* Our study shows that all logistics subsystems work on their capacity limits. The general skill shortage further increases this challenge, which is well-known in the logistics sector (Dubey et al. 2018). Recruiting and retaining qualified staff for warehouse operations and transportation gets increasingly demanding (Min and Lambert 2002; Min 2007). Skill shortage becomes particularly apparent in public holiday seasons when additional volumes have to be handled and further tasks have to be fulfilled under great time pressure.

Delivery capacities could be enlarged by shortening the delivery tours and increasing the utilization of trucks (see, e.g., Piecyk and McKinnon (2010)). This is, however, hard to manage due to increased traffic density, regulatory limitations and the already increased delivery volume in public holiday seasons. Our study also shows that the limited space in the warehouses and the lack of warehouse utilities leads to additional challenges in these periods.

The challenges associated with holidays share some similarities with situations considered in the literature on supply chain risk and resilience (Ivanov 2021a). Disruptions of the standard business with demand peaks and capacity shortages were recently observable during the Covid-19 pandemic in retailing and especially in grocery retailing (see, e.g., Schleper et al. (2021); Delasay et al. (2022)). The literature on supply chain risk management mainly distinguishes four types of uncertainties and risks (Klibi et al. 2010; Ivanov 2021a): First, random uncertainties, e.g., demand, price, and cost variation risks, which can be modeled and quantified using random variables with predictable probability distributions. Second, hazard uncertainty, i.e., the risk of an unusual event with a strong impact on one or more business functions, e.g., the loss of a key supplier. Third, deep uncertainty, i.e., a severe disruption risk, such as a natural disaster in a major supply and/or demand region. Fourth, a super-disruption event, i.e., a long-lasting crisis that affects multiple industries and impacts supply, production, demand, and logistics simultaneously, e.g., the Covid-19 pandemic. However, in contrast to the above-mentioned situations, the respective events and most of the influencing factors (weather, weekday constellation, etc.) are known in advance in the case of the public holiday challenge. Thus, the demand patterns of the respective peak periods as well as the respective availability of capacities and personnel can potentially be predicted relatively accurately. Compared to the situations and measures discussed in supply chain risk and resilience literature, a specific and detailed plan can be created for each situation considered. The greatest challenge here is to balance the extremely tight transportation capacities, the very limited availability of personnel, and the highly limited storage space in warehouses and outlets.

### Holiday logistics solutions in the light of literature

*Load profile.* Retailers shift workload in less utilized periods to balance the workload. That includes adjustments of delivery days, pre-picking and pre-delivery. These measures generally increase the in-store inventory level, even several weeks before public holidays. Inventory holding costs in grocery retailing, however, are typically less relevant than handling costs (van Donselaar et al. 2010). The retailers interviewed verify these observations. They intensively apply these measures, especially in Christmas and Easter business.

In grocery retailing workload balancing in the warehouse is commonly applied to smooth weekly seasonality (see, e.g., Vanheusden et al. (2020)). For public holiday seasons, balancing measures, however, have to be planned and executed over a longer period of time, to be able to smooth the exceptional workload peaks.

According to Rogerson and Santén (2017), balancing the transport volume in grocery retailing should be achieved by several actions like adjustments of the deliveries and order sizes. For standard weeks this can be done using IT systems and mathematical models (see, e.g., Holzapfel et al. (2016)). In public holiday weeks, however, many exceptional circumstances have to be considered and thus, tour planning is largely adapted manually.

*Resources.* According to van Gils et al. (2017), personnel resources are the main tool to handle seasonal fluctuations in warehouse operations. Similarly, personnel resources play a major role to handle seasonal peaks in transportation. Due to the problem of driver shortage, Min and Lambert (2002) identify factors increasing the satisfaction of drivers such as a competitive pay or the comfort of the truck. Motivation factors for warehouse staff include job satisfaction or a family-friendly working environment (Min 2007). Our interviewees confirm these literature reports. The retailers also offer employee-friendly working conditions and award their employees numerous benefits.

Wilding and Juriado (2004) note that retailers purchase external resources such as transport capacity from third parties if own resources are exhausted in public holiday seasons. Retailers apply this hybrid model to save costs and simultaneously assuring a certain degree of flexibility. Our survey, however, shows that most retailers interviewed prefer to maintain the responsibility for picking and delivery. They therefore establish long-term contracts with service providers that mostly allow flexible and short-term adaptations of resources required. Purchasing on spot markets is only considered as final possibility.

*Organization.* In the domain of organizational measures, automation plays a prominent role. Advanced forecasting technologies are becoming increasingly important for all logistics planning issues in grocery retailing. Accurate forecasts improve warehouse capacity management, workload balancing, and transport planning (Kim et al. 2018). Most retailers interviewed, however, struggle to apply automated forecasting techniques during public holiday seasons because of less suitable models and/or missing data. Retailers therefore manually adapt system-generated forecasts with great effort to improve forecast accuracy. Ehrenthal et al. (2014) already found that benefits can be generated when forecasts distinct between weekday and weekend figures. In addition, retailers should use data that depend on the planning horizon (Williams and Waller 2010). Our findings further indicate that weather forecasts, weekday constellations of holidays, and the stage of the selling seasons should be considered.

Furthermore, a higher degree of automation is becoming even more important for warehouse operations due to the lack of qualified personnel. The literature analyzes suitable robotics solutions supporting picking and packing (see, e.g., Azadeh et al. (2019)). Our findings further indicate that retailers that apply automated warehousing systems face similar challenges as their competitors, as automated systems so far are designed to work continuously at high utilization. That means, peaks in public holiday seasons can only be handled manually even though highly automated systems are available. Nevertheless, the grocery retailers interviewed see automation as an appropriate solution approach for the future.

Limiting the planning effort, retailers focus on pre-defined product groups during the disposition. Selection criteria are, for example, the extent of volatility due to their position in the store layout or little available data. Building product groups simplifies the forecasting process or enables forecasting for new products. This procedure in retail practice identified within our study is also suggested by several research papers. For example, Dekker et al. (2004) classify products with similar seasonal patterns for forecasting.

Moreover, retailers collaborate with up- and downstream supply chain actors to manage the public holiday challenge. Several papers analyze the pros and cons of retailer and supplier collaborations (see, e.g., Teller et al. (2016)). In our study we concrete the capability of retailer-supplier cooperation and show areas of application. A close supplier relationship for example reduces delivery failures and enables just-in-time deliveries that reduce the storage capacity required in the distribution center. In addition, retailers can support small suppliers with transport capacity in public holiday seasons.

Alftan et al. (2015) suggest collaborative forecasting for critical products in grocery retailing. Sharing sales and inventory figures is considered to be especially effective. We find that centralizing the planning tasks for the holiday business enhances the forecast quality at various retailers as all information is pooled and a central unit communicates with the supply chain partners.

Besides external cooperation, a harmonization of public holiday season plans of the different departments of a retailer (e.g., purchasing, logistics, marketing, sales) is required to allow efficient and effective public holiday operations. Collaboration of departments and the alignment of their planning is complex but improves the performance of logistics processes, resulting in less uncertainties, lower inventory levels or a higher availability (Dreyer et al. 2018). Although the participating retailers agree that close communication between departments is necessary, the implementation of measures is not generally executed in a collaborative way because of complexity reasons.

Again some of the approaches relevant for addressing the challenges associated with public holiday seasons show similarities to the approaches developed in the context of supply chain risk and resilience management that were recently particularly relevant to approach Covid-19 impacts in (grocery) retailing (see, e.g., Schleper et al. (2021) and Delasay et al. (2022)). Proactive and reactive (adaptation and recovery) measures can be taken to address these risks (Ivanov 2021b). The proposed proactive measures, i.e., creating flexibility or redundancy (see, e.g., Obermair et al. (2022)), show strong similarities to the approaches relevant to addressing the public holiday challenges. While these measures create general conditions for higher responsiveness, solution concepts for public holiday planning comprise additional mid-term capacity balancing elements that further relax workload in the peak seasons.

### Managerial implications

The sales divisions involved in the exploratory study cover around 80% of total market sales in German grocery retailing. This confirms the high relevance of the holiday issue in practice. 81% of study participants identify the Christmas season as one of the major challenges in grocery logistics. Due to the ban on working on public holidays and therefore the missing days for logistics, the typical concepts are not enough to handle all volume fluctuations in public holiday seasons. Therefore additional dedicated measures are necessary. Our study categorizes the solution approaches on a higher level and therefore highlights the links between all explored areas. The key to overcome the challenges in demand planning are IT systems. Data maintenance is therefore a decisive factor for forecasting quality. In addition, centralization of demand planning can be an efficient possibility for retailers as costs of separate planning of promotional items before public holidays can be kept to a minimum. Short-term reordering by stores can be avoided, as store-specific demand peaks can be predicted more accurately. Without short-term reorders, capacity requirements in the warehouse can be balanced on a mid-term basis by pre-picking and pre-delivery to the stores. Retailers may initiate these processes several weeks before the respective holiday seasons. This measure leads to an increase in inventory in stores, but frees up capacities in the distribution centers and transportation that are highly needed to deliver perishable groceries, fast moving, and high-value items during the public holiday weeks. Enabling this pre-working, extensive modifications of transportation tours are made that however pay off as transport capacities can be reserved on a reliable planning basis in advance which is relevant to overcome possible short-term transportation capacity scarcity or high costs on the spot market. Additionally, the early build-up of stock in the stores also balances the volume for refilling shelves and thus the tight and costly instore personnel capacity. However, the measures taken to reduce peak demand are generally not sufficient to satisfy the additional volume that occurs during public holiday weeks completely on a mid-term basis. Moreover, the balancing measures cannot be applied for perishable groceries, and are not efficient and cost-optimal for fast moving and high value items. Consequently, retailers need to expand their capacities in the distribution centers, transport and stores, and retain qualified staff, whose experience is highly demanded in the operationally complex and stressful peak seasons. Due to the scarcity of adequate personnel resources, retailers should offer an attractive working environment to avoid personnel fluctuation.

## Conclusion and further areas of research

*Conclusion.* Grocery retailers have to deal with the public holiday challenge several times per year. Nevertheless, literature so far only considered public holidays in a very general way and analyzed the corresponding logistics processes to a very limited extent. The present study provides insights into the operational planning issues in public holiday seasons. We analyze challenges and solution approaches of German grocery retailers for public holiday seasons, focusing on demand planning and disposition, warehouse management, and transportation management. Uncertainties and a shortage of resources mainly involve the challenges of public holiday seasons in grocery retailing. Retailers take up these challenges by a divers set of measures, which can be assigned to three general solution approaches: adjustment of workload profiles, adaptation of resources, and modifying organizations. Our findings disclose that retailers simultaneously adapt multiple processes in quite different logistics areas within public holiday seasons. This reveals the complexity and the interdependence of the occurring planning tasks. Until now the literature has not discussed this with comparable extent. Theory on retail operations management benefits from the structured overview on the divers challenges, their drivers and context-specifics, and the sets of solution concepts and their application conditions in retail practice. Retail practice on the other side benefits from our comprehensive overview of solution approaches proven to be adequate in the light of the individual challenges, which allows to rethink own approaches used. Retailers can thus leverage our findings to adequately develop public holiday logistics planning considering specific contexts. Our study shows that almost all retailers have room for considerable improvements.

*Further research.* Our study provides useful insights for research and practice of operational logistics planning and execution in grocery retailing. Nevertheless, there are numerous possibilities for future research.

1. While we have focused on mid- and short-term planning problems, future research may consider decisions with a longer planning horizon. A further study might address for example assortment or promotion planning and their respective implications for balancing volume peaks in the supply chain in public holiday seasons.
2. Further research could include the perspective of the upstream supply chain partners such as the suppliers and manufacturers. As noted in our findings section, there are numerous potentials for collaboration that can be further detailed and their impact on the companies’ performance could be analyzed.
3. Future research could focus on how automation techniques can support warehouse and transportation processes to compensate the general skill shortage.
4. Our study focuses on the German grocery market. Although we consider the findings as rather generic, future research could analyze other markets and compare the results to our findings.
5. Finally, future work can explore similar questions in other retail branches.

## Data Availability

The interview guideline of the exploratory study is included in the appendix. The interview results are presented in an anonymized and categorized manner within this paper. Individual data and company names are subject to confidentiality agreements and thus not presented within the paper.

## Appendix: Overview of interview guideline

### Standard processes

- How does the distribution of the picking volume during a week look like?
- Which flows of goods are common between the suppliers and the stores?
- How often do you deliver each product category to the stores?

### General insights on public holidays

- What are the major logistics challenges for grocery retailing before and after public holidays? Why are they challenging?
- Which planning areas are affected most by public holidays and why?
- How do public holidays influence the daily turnovers?

### Disposition

- Which problems occur in forecasting and disposition of public holiday seasons? Why are they challenging?
- Are there specific solution approaches to face disposition challenges in public holiday seasons? How do these approaches look like and what are the reasons behind?
- How are public holidays parameterized in your forecasting system?
- How do you adjust order quantities before public holidays? Why?
- How is the shift of the demand if a public holiday leads to a missing sales day?
- How do store disposition processes, e.g., order times, change in public holiday seasons?

### Warehouse planning

- Which problems occur in the warehouse in public holiday seasons? Why are they challenging?
- Are there specific solution approaches to face challenges in warehousing in public holiday seasons? How do these approaches look like and what are the reasons behind?
- How are holiday specific articles integrated in the warehouses?
- How do you deal with the reduced amount of potential picking days in public holiday weeks?
- Why have you designed your warehouse operations like this?
- Do you use stockpiling and how do you organize this with the stores?

### Transportation planning

- Which problems occur in the transport domain in public holiday seasons?
- Are there specific solution approaches to face transportation challenges in public holiday seasons? How do these approaches look like and what are the reasons behind?
- How do you execute the transport planning in public holiday seasons?
- Do you adjust tour plans, delivery rhythms, ramp management, etc.? How do you do this?
- Why have you chosen these instruments?
- How are internal store processes changed due to transportation?
